# Maternal asthma is associated with increased risk of perinatal mortality

**DOI:** 10.1371/journal.pone.0197593

**Published:** 2018-05-18

**Authors:** Mari Kemppainen, Anna-Maria Lahesmaa-Korpinen, Paula Kauppi, Martti Virtanen, Suvi M. Virtanen, Riitta Karikoski, Mika Gissler, Turkka Kirjavainen

**Affiliations:** 1 Department of Paediatrics, Children’s Hospital Helsinki University Hospital, Helsinki, Finland; 2 National Institute for Health and Welfare, Information Services Department, Unit of Statistics and Registers, Helsinki, Finland; 3 Helsinki University, Respiratory Medicine and Allergology, Helsinki, Finland; 4 Helsinki University Central Hospital, Skin and Allergy Hospital, Helsinki, Finland; 5 Nordic Casemix Center, Helsinki, Finland; 6 Unit of Nutrition, National Institute for Health and Welfare, Helsinki, Finland; 7 Health Sciences Center, University of Tampere, Center for Child Health Research, University of Tampere, Tampere, Finland; 8 Tampere University Hospital, and the Science Center of Pirkanmaa Hospital District, Tampere, Finland; 9 Regional State Administrative Agencies, Tampere, Finland; 10 Karolinska Institute, Department of Neurobiology, Care Sciences and Society, Division of Family Medicine, Stockholm, Sweden; University of Torino, ITALY

## Abstract

**Background:**

Asthma is the most common chronic disease during pregnancy and it may have influence on pregnancy outcome.

**Objectives:**

Our goal was to assess the association between maternal asthma and the perinatal risks as well as possible effects of asthma medication.

**Methods:**

The study was based on a nationwide Finnish register-based cohort between the years 1996 and 2012 in the Drug and Pregnancy Database. The register data comprised 962 405 singleton live and stillbirths, 898 333 (93.3%) pregnancies in mothers with neither confirmed asthma nor use of asthma medication (controls), and 26 674 (2.8%) pregnancies with confirmed maternal asthma. 71% of mothers with asthma used asthma medication. The diagnosis of asthma was based on the mothers’ right for subsidised medication which is carefully evaluated by strict criteria including pulmonary function testing. Odds ratio was used in comparison. Premature birth (PB), low birth weight, small for gestational age (SGA), neonatal death were the main outcome measures.

**Results:**

Maternal asthma was associated with adjusted odds ratios (aORs) for perinatal mortality 1.24 (95% CI 1.05 to 1.46), preterm birth 1.18 (1.11 to 1.25), low birth weight 1.29 (1.21 to 1.37), fetal growth restriction (SGA) 1.32, (1.24 to 1.40), and asphyxia 1.09 (1.02 to 1.17). Asthma treatment reduced the increased risk of preterm birth aOR 0.85 (95% CI 0.76 to 0.96) but mothers with treated asthma had higher risks of fetal growth restriction (SGA) aOR 1.26 (1.10 to 1.45), and asphyxia aOR 1.37 (1.17 to 1.61) than mothers with untreated asthma.

**Conclusion:**

Asthma is associated with increased risks of perinatal mortality, preterm birth, low birth weight, fetal growth restriction (SGA), and asphyxia. Asthma treatment reduces the risk of preterm delivery, but it does not seem to reduce other complications such as perinatal mortality.

## Introduction

Asthma is the most common chronic disease during pregnancy[1]. In Finland, the prevalence of physician diagnosed asthma has increased from six to ten percent from 1996 to 2006, asthma mortality and hospitalisation rates are low, and asthma treatment results are excellent in general [2,3].

The importance of public health questions on maternal asthma and asthma medication use during pregnancy has inspired several studies. However, previous cohort study findings based on single small cohorts on the relationships between maternal asthma and risk of perinatal complications have been conflicting [4–9]. By combining cohorts by meta-analysis, asthma has been shown to increase the risks of preeclampsia, preterm birth, low birth weight, fetal growth restriction (SGA), neonatal death, and congenital anomalies [10,11]. There are large cohort studies of mothers with asthma: three Swedish cohorts of 24 750, 26 586, and a recent cohort of 108 225, one US of 17,044, and one Canadian cohort of 13 100 mothers with asthma [4,12–16]. All these cohorts confirmed the association between maternal asthma and the risks of preterm birth and SGA. In addition, the Canadian cohort also showed an association between maternal asthma and increased perinatal mortality. Maternal asthma has been associated also with several mild neonatal complications such as hyperbilirubinemia and transient respiratory problems [10,11,14,17,18]. Based on small clinical studies, improved asthma management is suggested to decrease the asthma related increased perinatal risks[19–21].

We studied pregnancy complications in mothers with asthma in Finland based on complete, high quality registry data[22]. The data were acquired from the Drugs and Pregnancy database, which combines information from Finnish health registers. Our aim was to investigate the pregnancy complication risks associated with asthma and asthma medication.

## Methods

This is a national, population register based, cohort investigation including information on all the singleton live and stillbirths in Finland between 1996 and 2012. The data were acquired from the Finnish Drugs and Pregnancy database [23]. This database is a collaboration by THL National Institute for Health and Welfare, KELA Social Insurance Institution, and FIMEA Finnish Medicines Agency. The database contains medical and sociodemographic information on parents, pregnancy and perinatal outcomes, and reimbursed medication purchases. The linkage of registers has been approved by the data keepers (THL and KELA). An ethical committee has approved the formation of this database, and its utilisation in research studies of general interest. Since the registered women were not contacted, the ethical committee did not require informed consent from the study population. Each citizen and permanent resident of Finland has a personal identity code enabling data linkage between registers. All data were fully anonymized before analysis. Reporting to the official healthcare registers by public and private hospitals, and primary health care is mandatory by law.

The Medical Birth Register (MBR) was used to identify all the pregnancies and information on the outcomes. The registry data is recorded at the time of birth, and include all the live births and stillbirths (≥22 weeks of pregnancy or birth weight ≥500 grams).

The National Drug Reimbursement Register (DRR) contains individual-level data on reimbursed prescription medication purchases and data on the reimbursement for discounted medication for a restricted number of diseases. In Finland, all of the included asthma medications require a prescription for purchase. For asthma, the right for subsidised medication is granted according to strict criteria which include asthma symptoms, six months of regular medication use, and a reversible airway obstruction diagnostic for asthma in pulmonary function testing (Table 1). Once a person fulfils the criteria they are offered a right for subsidised medication, which is permanent for applicants over 16 years of age. In this study, the right for subsidised asthma medication indicates confirmed asthma. Asthma medication use was defined as asthma medication purchases during the period between three months prior the pregnancy and the end of the pregnancy. The three month period before the pregnancy was selected, because in Finland, the pharmacies are entitled to sell medications for up to three months at one time.

**Table 1:** The criteria used for the right for justification of reimbursement. For reimbursement, a person has to fulfil criteria in all three categories: symptoms, pulmonary function testing and medication purchase. Once a person fulfils the criteria they are offered reimbursement, which is permanent for applicants over 16 years of age.

**Physicians certificate–general requirements**
Typical development and clinical picture of asthma
Possible indicators of eosinophilic inflammation
Results of allergy test, pulmonary function tests and bronchial challenge tests
Persistent and ongoing symptoms of asthma for at least six months
Need for a controller medication for at least six months
**Pulmonary function testing–at least one of the following:**
Reversible obstruction during PEF monitoring of two weeks at least three times: daily variation of at least 20% and 60 l/min or improvement of PEF at least 15% and 60 l/min after bronchodilative medicine
In flow volume spirometry, an improvement of at least 12% and 200ml in FEV_1_ or FVC
After inhaled glucocorticoid treatment an increase of at least 15% and 200ml in FEV_1_ or increase in PEF level of 20%
Moderate or severe hyper responsiveness in bronchial challenge test to histamine or methacholine
A decrease of 15% or more in PEF or FEV_1_ in exercise test
**Medication purchases**
Regular use of asthma controller medication for at least six months from ATC-group R03AK (inhaled combination of β_2_ agonists and glucocorticoids) or R03BA (inhaled glucocorticoids) or (R03DC) leucotrienantagonist indicated by medication purchases is required

PEF = peak expiratory flow, FEV1 = forced expiratory volume in one second, FVC forced vital capacity

**Fig 1:**
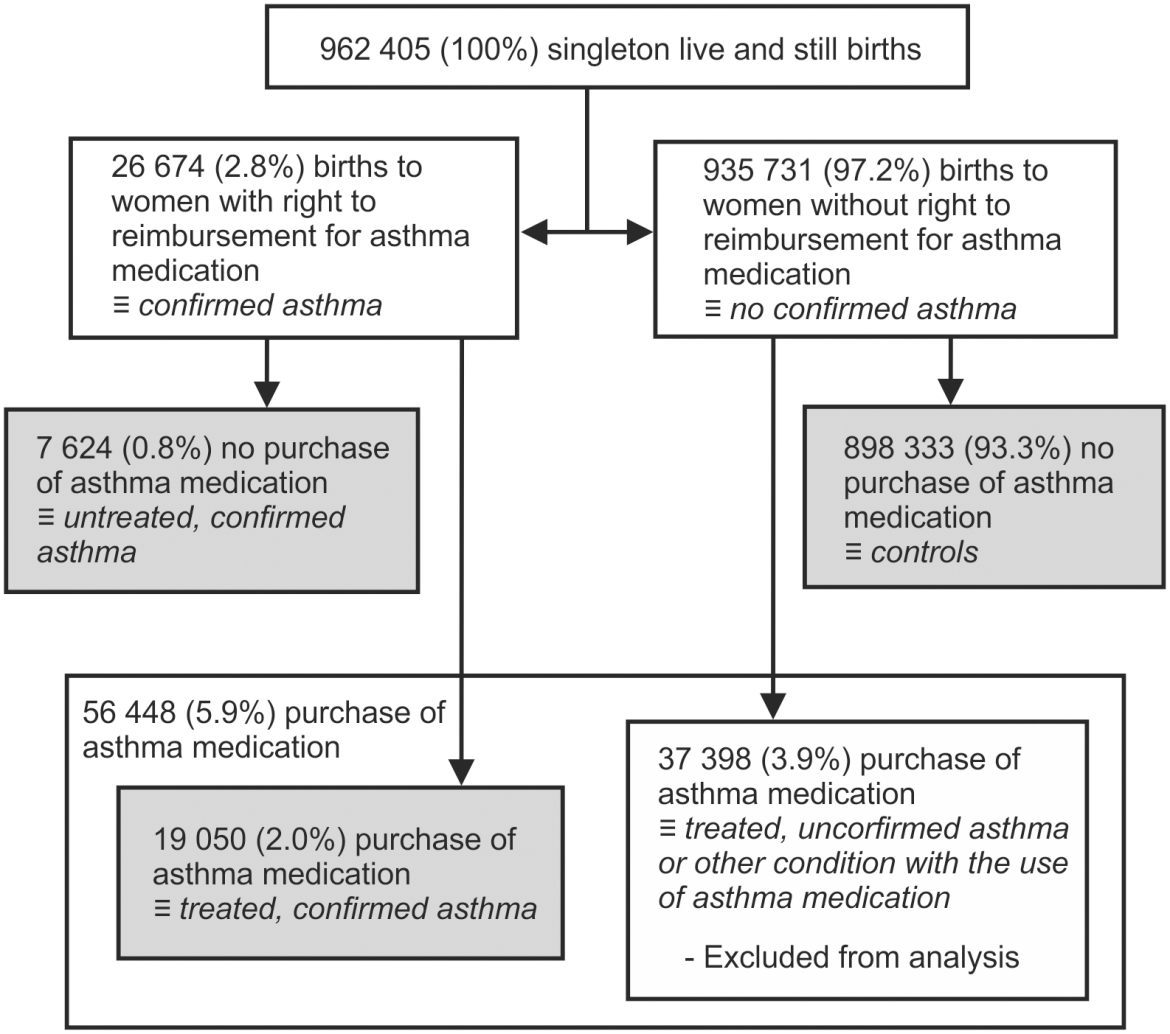
Demographics of the 962 405 singleton live and stillbirths between 1996 and 2012. The separation into different groups.

For comparisons, we identified three groups of mothers: 1) controls as mothers with neither confirmed asthma nor current use of asthma medication, 2) untreated asthma as mothers with confirmed asthma but no use of asthma medication, and 3) treated asthma as mothers with confirmed asthma and use of asthma medication (Fig 1). Also, a fourth group was recognised as mothers without confirmed asthma but use of asthma medication. This group was excluded from analysis (Fig 1).

For study purposes, the Anatomical Therapeutic Chemical (ATC) grouping was used, and the following categories were identified: inhaled short- and long-acting β_2_ agonists (R03AC), inhaled glucocorticoids (R03BA), inhaled combination of β_2_ agonists and glucocorticoids (R03AK), inhaled short- and long-acting anticholinergics (R03BB), xanthines (R03DA), leukotriene receptor antagonists (R03DC), and oral glucocorticoids (H02AB).

The primary outcomes of this study were preterm birth (<37 weeks), low birth weight (<2500 grams), perinatal mortality (stillbirths and early neonatal deaths), and small for gestational age (defined by population based sex-specific references).[24] In perinatology, the length of the pregnancy and perinatal mortality are the most commonly used core outcomes of pregnancy.[25]

The secondary outcomes were umbilical cord arterial pH <7.10, Apgar score <7, and urgent section. Apgar score is a well-established classification system of newborn health. The 5 minute Apgar scores were available in the Medical Birth Register since 2004. The asphyxia diagnose is made postnatally from the arterial or venous cord pH and Apgar score, or prenatally from cardiotocogram. The information regarding urgent sections was selected as an outcome measure to represent an observed consequence of the fetal distress and asphyxia. Urgent section takes place within 30 minutes on section decision.

The used confounding factors were maternal age, smoking while pregnant, socioeconomic status based on maternal occupation during pregnancy, and parity. As the reported prevalence of epilepsy did not differ between the maternal asthma groups, and as the amount of mothers with type 1 and 2 diabetes was small, these factors were not used for adjusting.

Differences between different exposure groups were evaluated by chi-squared test for dichotomous and categorical variables. Adjusted odds ratios (aOR) and 95% confidence intervals (CI) were estimated to measure all associations, using unconditional logistic regression analysis. Possible confounding factors were selected based on the literature (see above). Individuals with missing outcome variables were excluded from the analysis.

## Results

The demographic data and criteria for confirmed asthma diagnosis are presented in Fig 1 and Table 1. Supplemental S1 Table shows characteristics of mothers in detail. The study cohort included of 962 405 live and stillbirths, 898 333 (93.3%) control mothers and 26 674 (2.8%) mothers with confirmed asthma, and 5 292 perinatal deaths. For subgroup analysis, asthmatic mothers were subdivided into 19 059 (2.0%) mothers with treated confirmed asthma, and 7 624 (0.8%) mothers with untreated confirmed asthma. Of the mothers with treated confirmed asthma, 64.6% used at least two asthma medications belonging to separate ATC-groups.

The main study results are presented in Table 2, and S2 and S3 Tables. S3 Table represents all adjusted and unadjusted odds ratios. Compared to control pregnancies, the infants born to mothers with asthma showed increased risk of perinatal mortality, preterm birth, low birth weight, fetal growth restriction (SGA), umbilical arterial pH below 7.1, Apgar score below 7 at 1 minutes and 5 minutes of age, delivery by urgent section, and birth asphyxia. In a subgroup analysis comparing pregnancies between mothers with asthma, asthma treatment reduces the increased risk of preterm birth. However, mothers with treated asthma had higher risks of fetal growth restriction (SGA) and asphyxia than mothers with untreated asthma.

**Table 2:** Perinatal outcomes in mothers with confirmed, treated or untreated asthma for singleton live and stillbirths.

	Confirmed asthma vs control		Treated vs. untreated. confirmed asthma		Untreated. confirmed asthma vs control		Treated. confirmed asthma vs. control	
	aOR* (95% CI)	*P*	aOR* (95% CI)	*P*	aOR* (95% CI)	*P*	aOR* (95% CI)	*P*
Perinatal mortality	1.26 (1.07, 1.48)	0.0067	0.93 (0.66, 1.32)	0.6903	1.33 (1.00, 1.79)	0.0533	1.22 (1.01, 1.49)	0.0447
Premature birth	1.18 (1.12, 1.25)	<0.0001	0.87 (0.77, 0.98)	0.0210	1.31 (1.19, 1.45)	<0.0001	1.13 (1.05, 1.21)	0.0005
Low birth weight	1.29 (1.21, 1.37)	<0.0001	1.12 (0.97, 1.29)	0.1144	1.19 (1.05, 1.34)	0.0058	1.33 (1.23, 1.43)	<0.0001
SGA	1.32 (1.24, 1.40)	<0.0001	1.25 (1.09, 1.44)	0.0015	1.12 (0.99, 1.26)	0.0691	1.39 (1.30, 1.49)	<0.0001
Umbilical arterial pH < 7.1	1.17 (1.08, 1.26)	<0.0001	1.10 (0.92, 1.31)	0.3027	1.10 (0.94, 1.28)	0.2449	1.20 (1.09, 1.31)	0.0001
1 min Apgar score 0 to 6	1.26 (1.19, 1.32)	<0.0001	1.01 (0.91, 1.14)	0.8172	1.24 (1.13, 1.36)	<0.0001	1.26 (1.19, 1.34)	<0.0001
5 min Apgar score 0 to 6	1.21 (1.09, 1.35)	0.0005	1.05 (0.82, 1.35)	0.6829	1.15 (0.93, 1.43)	0.1934	1.23 (1.09, 1.40)	0.0009
Urgent section	1.23 (1.18, 1.28)	<0.0001	1.12 (1.03, 1.23)	0.0119	1.13 (1.05, 1.23)	0.0019	1.27 (1.21, 1.33)	<0.0001
Asphyxia	1.07 (1.00, 1.14)	0.0454	1.32 (1.12, 1.55)	0.0007	0.88 (0.77, 1.01)	0.0774	1.14 (1.06, 1.23)	0.0007

* Adjusted for maternal age, parity, smoking, socio-economic status, year of birth

Table 3 and S4 Table present risk factors associated with each medication type and their combinations in pregnancies. The use of short-acting β_2_ agonists was associated with increased perinatal mortality, but on the other hand, 71% of mothers with treated asthma used these short-acting β_2_ agonists, and the increased risk of perinatal mortality could be a result of the underlying disease. The number of used asthma medications increased the risk of low birth weight and fetal growth restriction in logistic regression analysis on 0, 1, 2, and 3 or more medications. Each purchased medication group increased the risk of low birth weight by 6% aOR 1.06 (95% CI 1.00 to 1.13, *P = 0* .0356), and fetal growth restriction (SGA) by 13% aOR 1.13 (95% CI 1.07 to 1.19, *P* < 0.0001). However, the risk of perinatal mortality or preterm birth did not increase in logistic manner.

**Table 3:** Perinatal outcomes of treated, confirmed asthma by ATC-groups compared to controls for singleton live and stillbirths.

	Perinatal mortality		Premature birth		Low birth weight		SGA	
	aOR* (95% CI)	*P*	aOR* (95% CI)	*P*	aOR* (95% CI)	*P*	aOR* (95% CI)	*P*
Any asthmamedication	1.22 (1.01, 1.49)	0.0447	1.13 (1.05, 1.21)	0.0005	1.33 (1.23, 1.43)	<0.0001	1.39 (1.30, 1.49)	<0.0001
No asthma medication	1.33 (1.00, 1.79)	0.0533	1.31 (1.19, 1.45)	<0.0001	1.19 (1.05, 1.34)	0.006	1.12 (0.99, 1.26)	0.069
One asthma medication	1.29 (0.92, 1.80)	0.1477	1.19 (1.05, 1.33)	0.0048	1.21 (1.06, 1.39)	0.004	1.19 (1.05, 1.36)	0.009
Two asthma medications	1.23 (0.92, 1.64)	0.1601	1.06 (0.96, 1.18)	0.2557	1.37 (1.24, 1.52)	<0.0001	1.46 (1.32, 1.61)	<0.0001
Three or more asthma medications	1.12 (0.74, 1.71)	0.5859	1.18 (1.03, 1.36)	0.0168	1.39 (1.20, 1.60)	<0.0001	1.53 (1.33, 1.75)	<0.0001

* Adjusted for maternal age, parity, smoking, socio-economic status, year of birth

## Discussion

### Main findings

This comprehensive register based study shows that confirmed maternal asthma is associated with the risk of perinatal mortality with the adjusted odds ratio of 1.24 (95% CI 1.05 to 1.46). In addition, like previously shown, maternal asthma is associated with increased risks of preterm birth, low birth weight, fetal growth restriction (SGA), and asphyxia. When comparing treated and untreated group of mothers with asthma, there was no significant difference in perinatal mortality between the groups, but asthma treatment decreased the increased risk of preterm birth. However, the risks of SGA and asphyxia remained higher in mothers with treated asthma.

### Strengths and limitations

The major strengths of this study are national coverage of the two obligatory registers, the large size of the study cohort, the strict criteria applied for the diagnosis of persistent asthma, the exclusion of the use of any asthma medication in the group of control mothers, and possibility to control several confounding factors such as parity, maternal age, socioeconomic status, self-reported smoking, and epilepsy.[22] In this study, we have been able to cover some of the possible weaknesses of previous three published large cohort studies on this subject. The Swedish study by Källén and associates was based on self-reported use of asthma medication on maternity care visit between 10 and 12 weeks of pregnancy [4,17,26]. The US cohort was based on asthma diagnosis (ICD-9) in patient records, in well-selected hospitals across US and contained no information on use of asthma medications [14,15,27]. The Canadian cohort was based on purchases of asthma medication of substantial but not whole population of one province in Canada [16].

Our cohort included 26 674 (2.8%) children born to mothers with confirmed asthma, and 71% of these mothers used asthma medication. We estimated the use of medication through the medicine reimbursement registry with full coverage of medications purchased in Finland. Although not confirmed, we assume that asthma medication purchase reliably correlates to the real use of medication, and thus serves well for the study purposes.

The limitations of this study are that from this registry data, we were unable to separate different asthma phenotypes such as allergic and non-allergic asthma or dosing of medication. Like none of the cohort studies so far, we were not able to control the severity of maternal asthma through pulmonary function testing or internationally validated test of asthma control [28].

The data on asthma diagnosis was based on reimbursement records of the national Drug Reimbursement Register. The criteria for the right for subsidised medication are a key element when interpreting our results (Table 1). The use of strict criteria through medical reimbursement guidelines imply that a significant number of mothers with some degree of asthma were not included in the group of treated confirmed asthma. The results that 37 398 (3.9%) of mothers had purchased asthma medications without the right for subsidised asthma medication support this assumption. These mothers were likely to have asthma symptoms but had either no objective verification of airway reversibility or the need for previous regular asthma medication for more than six months. The exclusion of mothers with intermittent asthma medication use made it possible to compare the fetal effects of asthma itself irrespective of asthma medication. The criteria for asthma used in Finland are according to international asthma guidelines, making the study generalizable [29–31].

### Interpretation

Since asthma symptoms may vary over time, we assumed that most mothers with untreated asthma were currently asymptomatic, and therefore, without purchases of asthma medication despite previously diagnosed asthma. However, we cannot exclude poor adherence to asthma treatment. Nevertheless, we suggest that the observed association with the risk of perinatal mortality in this group is caused by the asthma process itself.

In the meta-analysis by Murphy and associates, maternal asthma increased the risk of neonatal death [10]. In the single large Canadian cohort, maternal asthma increased the risk of perinatal mortality in general, but this correlation vanished after adjustment for preterm birth and SGA [16]. These US and Canadian cohorts were not able to differentiate effects of asthma treatment on the risk analysis. Källén and associates showed in the Swedish cohort of 24 369 births to mothers with asthma, that the use of three or more asthma medications indicating severe asthma increased the risk of perinatal mortality [4]. The Swedish cohort was not able to differentiate untreated asthma or oral glucocorticoid treatment, and they did not have coverage of medication prescriptions for the whole pregnancy. In our cohort, unlike in the study of Källén, the use of increasing number of asthma medications did not significantly increase the risk of perinatal mortality [17].

Our data showed that asthma medication diminishing the risk of preterm birth but not the increased risks of SGA or perinatal death. This observation is supported by Rejnö and associates in newer Swedish cohorts [12,13].

According to the current asthma guidelines, asthma medications are recommended to be used in a stepwise manner [29–31]. The number of used asthma medications or the use of did not correlate with the increased risk of perinatal mortality or preterm birth but it did correlate with low birth weight and SGA. We suggest that these correlations with low birth weight and SGA reflect the severity of asthma more than the drug effect. Schatz and associates showed, that the severity of asthma during pregnancy determined by spirometry adjusted for the use of oral glucocorticoids increased the risk of preterm birth [32]. In the large Canadian cohort reported by Cossette and associates, the use of a combination of low to moderate doses of inhaled corticosteroids and long-acting β_2_ agonists adjusted for asthma severity did not associate with perinatal risks [33].

In the future, we will analyse the effect of intermittent asthma-medication use, dosage, the use of asthma medication in different trimesters of pregnancy, and consider of combining registry based data with other available information such as medical records.

## Conclusion

Asthma is frequently encountered in pregnant women. Asthma is associated with increased risk of perinatal mortality, preterm birth, low birth weight, fetal growth restriction (SGA), and asphyxia. Asthma treatment reduces the increased risk for preterm delivery but it does not seem to reduce the risk of perinatal mortality and other complications. The mechanisms remain elusive. The entire causal pathway from maternal asthma to adverse perinatal outcomes especially growth retardation may not be targeted by current medications for asthma.

## Supporting information

S1 Table Characteristics of the mothers. The study consisted of 962 405 singleton live and stillbirths in Finland between the years 1996 and 2012.
(DOC)

S2 Table Frequency of perinatal outcomes. The study consisted of 962 405 live- and stillborn singletons in Finland between the years 1996 and 2012, Chi-square test between groups.
(DOC)

S3 Table Perinatal outcomes in mothers with confirmed, treated or untreated asthma for singleton live and stillbirths. Unadjusted and adjusted odds ratios.
(DOC)

S4 Table Perinatal outcomes of treated, confirmed asthma by ATC-groups compared to controls for singleton live and stillbirths. (DOC)

## References

[1] Kwon HL, Triche EW, Belanger K, Bracken MB. The epidemiology of asthma during pregnancy: prevalence, diagnosis, and symptoms. Immunol Allergy Clin North Am. 2006;26: 29–62. doi: 10.1016/j.iac.2005.11.002 16443142 10.1016/j.iac.2005.11.002

[2] Kauppi P, Linna M, Martikainen J, Mäkelä MJ, Haahtela T. Follow-up of the Finnish Asthma Programme 2000–2010: reduction of hospital burden needs risk group rethinking. Thorax. 2013;68: 292–293. doi: 10.1136/thoraxjnl-2011-201028 22504963 10.1136/thoraxjnl-2011-201028

[3] Kainu A, Pallasaho P, Piirilä P, Lindqvist A, Sovijärvi A, Pietinalho A. Increase in prevalence of physician-diagnosed asthma in Helsinki during the Finnish Asthma Programme: improved recognition of asthma in primary care? A cross-sectional cohort study. Prim Care Respir J. 2013;22: 64–71. doi: 10.4104/pcrj.2013.00002 23299455 10.4104/pcrj.2013.00002 PMC6442759

[4] Källén B, Otterblad Olausson P. Use of anti-asthmatic drugs during pregnancy. 1. Maternal characteristics, pregnancy and delivery complications. Eur J Clin Pharmacol. 2007;63: 363–373. doi: 10.1007/s00228-006-0257-1 17265060 10.1007/s00228-006-0257-1

[5] Bracken MB, Triche EW, Belanger K, Saftlas A, Beckett WS, Leaderer BP. Asthma symptoms, severity, and drug therapy: a prospective study of effects on 2205 pregnancies. Obstet Gynecol. 2003;102: 739–752. 14551004 10.1016/s0029-7844(03)00621-5

[6] Alexander S, Dodds L, Armson BA. Perinatal outcomes in women with asthma during pregnancy. Obstet Gynecol. 1998;92: 435–440. 9721785 10.1016/s0029-7844(98)00191-4

[7] Stenius-Aarniala BS, Hedman J, Teramo KA. Acute asthma during pregnancy. Thorax; 1996;51: 411–414. doi: 10.1136/thx.51.4.411 8733495 10.1136/thx.51.4.411 PMC1090678

[8] Perlow JH. Severity of asthma and perinatal outcome. 1992;167: 963–967. 10.1016/s0002-9378(12)80020-2 1415433

[9] Olesen C, Thrane N, Nielsen GL, Sørensen HT, Olsen J, EuroMAP Group. A population-based prescription study of asthma drugs during pregnancy: changing the intensity of asthma therapy and perinatal outcomes. Respiration. 2001;68: 256–261. doi: 10.1159/000050507 11416245 10.1159/000050507

[10] Murphy VE, Wang G, Namazy JA, Powell H, Gibson PG, Chambers C, et al The risk of congenital malformations, perinatal mortality and neonatal hospitalisation among pregnant women with asthma: a systematic review and meta-analysis. BJOG. 2013;120: 812–822. doi: 10.1111/1471-0528.12224 23530780 10.1111/1471-0528.12224

[11] Murphy V. A meta-analysis of adverse perinatal outcomes in women with asthma. BJOG.; 2011;118: 1314–1323. doi: 10.1111/j.1471-0528.2011.03055.x 21749633 10.1111/j.1471-0528.2011.03055.x

[12] Rejnö G, Lundholm C, Larsson K, Larsson H, Lichtenstein P, D’Onofrio BM, et al Adverse Pregnancy Outcomes in Asthmatic Women: A Population-Based Family Design Study. J Allergy Clin Immunol Pract. 2017 doi: 10.1016/j.jaip.2017.07.036 28988783 10.1016/j.jaip.2017.07.036

[13] Rejnö G, Lundholm C, Gong T, Larsson K, Saltvedt S, Almqvist C. Asthma during pregnancy in a population-based study—pregnancy complications and adverse perinatal outcomes. Laine K, editor. PLoS ONE. 2014;9: e104755 doi: 10.1371/journal.pone.0104755 25141021 10.1371/journal.pone.0104755 PMC4139314

[14] Mendola P, Männistö TI, Leishear K, Reddy UM, Chen Z, Laughon SK. Neonatal health of infants born to mothers with asthma. Journal of Allergy and Clinical Immunology. 2014;133: 85–90.e4. doi: 10.1016/j.jaci.2013.06.012 23916153 10.1016/j.jaci.2013.06.012 PMC3874245

[15] Mendola P, Laughon SK, Männistö TI, Leishear K, Reddy UM, Chen Z, et al Obstetric complications among US women with asthma. Am J Obstet Gynecol. 2013;208: 127.e1–8. doi: 10.1016/j.ajog.2012.11.007 23159695 10.1016/j.ajog.2012.11.007 PMC3557554

[16] Breton M-C, Beauchesne M-F, Lemière C, Rey E, Forget A, Blais L. Risk of perinatal mortality associated with asthma during pregnancy. Thorax. 2009;64: 101–106. doi: 10.1136/thx.2008.102970 19008298 10.1136/thx.2008.102970

[17] Källén B, Otterblad Olausson P. Use of anti-asthmatic drugs during pregnancy. 2. Infant characteristics excluding congenital malformations. Eur J Clin Pharmacol. 2007;63: 375–381. doi: 10.1007/s00228-006-0258-0 17265059 10.1007/s00228-006-0258-0

[18] Demissie K, Marcella SW, Breckenridge MB, Rhoads GG. Maternal asthma and transient tachypnea of the newborn. Pediatrics. 1998;102: 84–90. 9651418 10.1542/peds.102.1.84

[19] Stenius-Aarniala B, Piirila P, Teramo K. Asthma and pregnancy: a prospective study of 198 pregnancies. Thorax. 1988;43: 12–18. doi: 10.1136/thx.43.1.12 2895502 10.1136/thx.43.1.12 PMC461079

[20] Powell H, Murphy VE, Taylor DR, Hensley MJ, McCaffery K, Giles W, et al Management of asthma in pregnancy guided by measurement of fraction of exhaled nitric oxide: a double-blind, randomised controlled trial. Lancet. 2011;378: 983–990. doi: 10.1016/S0140-6736(11)60971-9 21907861 10.1016/S0140-6736(11)60971-9

[21] Tamási L, Bohács A, Horváth I, Losonczy G. Asthma in pregnancy—from immunology to clinical management. Multidiscip Respir Med. BioMed Central Ltd; 2010;5(4):259–63. 10.1186/2049-6958-5-4-259 PMC3436629 22958582

[22] Gissler M, Teperi J, Hemminki E, Meriläinen J. Data quality after restructuring a national medical registry. Scand J Soc Med. 1995;23: 75–80. 7784857 10.1177/140349489502300113

[23] Artama M, Gissler M, Malm H, Ritvanen A, Drugs and Pregnancy Study Group. Nationwide register-based surveillance system on drugs and pregnancy in Finland 1996–2006. Pharmacoepidemiol Drug Saf. 2011;20: 729–738. doi: 10.1002/pds.2159 21626607 10.1002/pds.2159

[24] Sankilampi U, Hannila M-L, Saari A, Gissler M, Dunkel L. New population-based references for birth weight, length, and head circumference in singletons and twins from 23 to 43 gestation weeks. Ann Med. 2013;45: 446–454. doi: 10.3109/07853890.2013.803739 23768051 10.3109/07853890.2013.803739

[25] Meher S, Alfirevic Z. Choice of primary outcomes in randomised trials and systematic reviews evaluating interventions for preterm birth prevention: a systematic review. BJOG. 2014;121: 1188–94– discussion 1195–6. doi: 10.1111/1471-0528.12593 24571433 10.1111/1471-0528.12593

[26] Källén B, Otterblad Olausson P. Use of anti-asthmatic drugs during pregnancy. 3. Congenital malformations in the infants. Eur J Clin Pharmacol. 2007;63: 383–388. doi: 10.1007/s00228-006-0259-z 17279357 10.1007/s00228-006-0259-z

[27] Zhang J, Troendle J, Reddy UM, Laughon SK, Branch DW, Burkman R, et al Contemporary cesarean delivery practice in the United States. Am J Obstet Gynecol. 2010;203: 326.e1–326.e10. doi: 10.1016/j.ajog.2010.06.058 20708166 10.1016/j.ajog.2010.06.058 PMC2947574

[28] Palmsten K, Schatz M, Chan PH, Johnson DL, Chambers CD. Validation of the Pregnancy Asthma Control Test. J Allergy Clin Immunol Pract. 2016;4: 310–5.e1. doi: 10.1016/j.jaip.2015.11.019 26776373 10.1016/j.jaip.2015.11.019 PMC4789157

[29] Society BT. British guideline on the management of asthma https://www.brit-thoracic.org.uk/standards-of-care/guidelines/btssign-british-guideline-on-the-management-of-asthma/ Visitide at March 2018. In: https://www.brit-thoracic.org.uk/standards-of-care/guidelines/btssign-british-guideline-on-the-management-of-asthma/. Sep 2016 p. 214.

[30] Chung KF, Wenzel SE, Brozek JL, Bush A, Castro M, Sterk PJ, et al International ERS/ATS guidelines on definition, evaluation and treatment of severe asthma. Eur Respir J. 2014;43: 343–373. doi: 10.1183/09031936.00202013 24337046 10.1183/09031936.00202013

[31] Asthma GIF. Global strategy for asthma management and prevention, update 2017 http://ginasthma.org/2017-gina-report-global-strategy-for-asthma-management-and-prevention/. In: http://ginasthma.org/2017-gina-report-global-strategy-for-asthma-management-and-prevention/.

[32] Schatz M, Dombrowski MP, Wise R, Momirova V, Landon M, Mabie W, et al Spirometry is related to perinatal outcomes in pregnant women with asthma. Am J Obstet Gynecol. 2006;194: 120–126. doi: 10.1016/j.ajog.2005.06.028 16389020 10.1016/j.ajog.2005.06.028

[33] Cossette B, Forget A, Beauchesne M-F, Rey É, Lemière C, Larivée P, et al Impact of maternal use of asthma-controller therapy on perinatal outcomes. Thorax. 2013;68: thoraxjnl–2012–203122–730. doi: 10.1136/thoraxjnl-2012-203122 23585516 10.1136/thoraxjnl-2012-203122

