# Supplementary material for: Maternal asthma is associated with increased risk of perinatal mortality
Source: PLoS One. 2018 May 18;13(5):e0197593. doi: 10.1371/journal.pone.0197593 (PMC5959067; doi:10.1371/journal.pone.0197593)
Supplement: S1 Table — The study consisted of 962 405 singleton live and stillbirths in Finland between the years 1996 and 2012. (DOC) [file pone.0197593.s001.doc]

**Table S1. Characteristics of the mothers. The study consisted of 962 405 singleton live and stillbirths in Finland between the years 1996 and 2012, Chi-square test between groups.**

|  | **Total** | | **Control** | | **Confirmed asthma** | | **Untreated confirmed asthma** | | **Treated confirmed asthma** | | **p** |
| --- | --- | --- | --- | --- | --- | --- | --- | --- | --- | --- | --- |
|  | **N** | **%** | **N** | **%** | **N** | **%** | **N** | **%** | **N** | **%** |  |
| **Number** | 962 405 | 100 ^a^ | 898 333 | 93.3 ^a^ | 26 674 | 2.8 ^a^ | 7 624 | 0.8 ^a^ | 19 050 | 2.0 ^a^ |
| **Asthma medication** | | | | | | | | | | | |
| Any asthma medication | 56 448 | 5.9 ^a^ | 0 | 0 | 19 050 | 71.4 ^c^ | 0 | 0 | 19 050 | 100 ^e^ |  |
| At least two asthma medications from different ATC-groups | 23 614 | 2.5 ^a^ | 0 | 0 | 12 985 | 48.7 ^c^ | 0 | 0 | 12 985 | 68.2 ^e^ |
| **Maternal age at labour** | | | | | | | | | | | |
| <20 | 25 453 | 2.6 ^a^ | 23 926 | 2.7 ^b^ | 502 | 1.9 ^c^ | 177 | 2.3 ^d^ | 325 | 1.7 ^e^ | <0.001 |
| 20-34 | 761 336 | 79.1 ^a^ | 712 033 | 79.3 ^b^ | 20 574 | 77.1 ^c^ | 5 917 | 77.6 ^d^ | 14 657 | 76.9 ^e^ |
| ≥35 | 175 616 | 18.3 ^a^ | 162 374 | 18.1 ^b^ | 5 598 | 21.0 ^c^ | 1 530 | 20.1 ^d^ | 4 068 | 21.4 ^e^ |
| **Parity** | | | | | | | | | | | |
| Primiparous | 398 694 | 41.4 ^a^ | 372 051 | 41.4 ^b^ | 10 670 | 40.0 ^c^ | 2 742 | 36.0 ^d^ | 7 928 | 41.6 ^e^ | <0.001 |
| Multiparous | 562 158 | 58.4 ^a^ | 524 816 | 58.4 ^b^ | 15 968 | 59.9 ^c^ | 4 873 | 63.9 ^d^ | 11 095 | 58.2 ^e^ |
| Unknown | 1 553 | 0.2 ^a^ | 1 466 | 0.2 ^b^ | 36 | 0.1 ^c^ | 9 | 0.1 ^d^ | 27 | 0.1 ^e^ |
| **Socioeconomic class based on occupation** | | | | | | | | | | | |
| Managerial employee | 163 918 | 17.0 ^a^ | 153 077 | 17.0 ^b^ | 4 467 | 16.7 ^c^ | 1 239 | 16.3 ^d^ | 3 228 | 16.9 ^e^ | <0.001 |
| Employee | 341 588 | 35.5 ^a^ | 317 768 | 35.4 ^b^ | 10 795 | 40.5 ^c^ | 3 067 | 40.2 ^d^ | 7 728 | 40.6 ^e^ |
| Worker | 136 584 | 14.2 ^a^ | 128 840 | 14.3 ^b^ | 3 069 | 11.5 ^c^ | 895 | 11.7 ^d^ | 2 174 | 11.4 ^e^ |
| Student | 89 378 | 9.3 ^a^ | 83 437 | 9.3 ^b^ | 2 410 | 9.0 ^c^ | 705 | 9.3 ^d^ | 1 705 | 9.0 ^e^ |
| Entrepreneur | 16 417 | 1.7 ^a^ | 15 277 | 1.7 ^b^ | 451 | 1.7 ^c^ | 140 | 1.8 ^d^ | 311 | 1.6 ^e^ |
| Other | 59 031 | 6.1 ^a^ | 55 232 | 6.2 ^b^ | 1 674 | 6.3 ^c^ | 538 | 7.1 ^d^ | 1 136 | 6.0 ^e^ |
| Unknown | 155 489 | 16.2 ^a^ | 144 702 | 16.1 ^b^ | 3 808 | 14.3 ^c^ | 1 040 | 13.6 ^d^ | 2 768 | 14.5 ^e^ |
| **Smoking while pregnant** | | | | | | | | | | | |
| Did not smoke | 793 369 | 82.6 ^a^ | 741 928 | 82.8 ^b^ | 22 040 | 82.6 ^c^ | 6 236 | 82.0 ^d^ | 15 804 | 83.1 ^e^ | 0.489 |
| Quit smoking after 1st trimester | 32 556 | 3.4 ^a^ | 30 124 | 3.4 ^b^ | 873 | 3.3 ^c^ | 271 | 3.6 ^d^ | 602 | 3.2 ^e^ |
| Smoked after1st trimester | 111 508 | 11.6 ^a^ | 102 890 | 11.5 ^b^ | 3 094 | 11.6 ^c^ | 898 | 11.8 ^d^ | 2 196 | 11.5 ^e^ |
| Unknown | 22 944 | 2.4 ^a^ | 21 486 | 2.4 ^b^ | 623 | 2.3 ^c^ | 201 | 2.6 ^d^ | 422 | 2.2 ^e^ |
| **Diabetes and epilepsy** | | | | | | | | | | | |
| Diabetes | 5 128 | 0.5 ^a^ | 4 725 | 0.5 ^b^ | 182 | 0.7 ^c^ | 67 | 0.9 ^d^ | 115 | 0.6 ^e^ | <0.001 |
| Epilepsy | 6 599 | 0.7 ^a^ | 6 114 | 0.7 ^b^ | 183 | 0.7 ^c^ | 64 | 0.8 ^d^ | 119 | 0.6 ^e^ | 0.291 |
| ^a^= percentage from total, ^b^= percentage from controls, ^c^= percentage from mothers with confirmed asthma, ^d^=percentage from mothers with untreated, confirmed asthma, ^e^=percentage from mothers with treated, confirmed asthma | | | | | | | | | | |  |
