# Supplementary material for: Maternal asthma is associated with increased risk of perinatal mortality
Source: PLoS One. 2018 May 18;13(5):e0197593. doi: 10.1371/journal.pone.0197593 (PMC5959067; doi:10.1371/journal.pone.0197593)
Supplement: S2 Table — The study consisted of 962 405 live- and stillborn singletons in Finland between the years 1996 and 2012, Chi-square test between groups. (DOC) [file pone.0197593.s002.doc]

**Table S2. Frequency of perinatal outcomes. The study consisted of 962 405 live- and stillborn singletons in Finland between the years 1996 and 2012.**

|  | **Total** | | **Control** | | **Confirmed asthma** | | **Untreated confirmed asthma** | | **Treated confirmed asthma** | |
| --- | --- | --- | --- | --- | --- | --- | --- | --- | --- | --- |
|  | **N** | **%** | **N** | **%** | **N** | **%** | **N** | **%** | **N** | **%** |
| Perinatal mortality | 4 482 | 0.5 ^a^ | 4 152 | 0.5 ^b^ | 152 | 0.6 ^c^ | 48 | 0.6 ^d^ | 104 | 0.6 ^e^ |
| Missing | 0 | 0.0 ^a^ | 0 | 0.0 ^b^ | 0 | 0.0 ^c^ | 0 | 0.0 ^d^ | 0 | 0.0 ^e^ |
| Premature birth | 40 635 | 4.2 ^a^ | 37 456 | 4.2 ^b^ | 1 301 | 4.9 ^c^ | 407 | 5.3 ^d^ | 894 | 4.7 ^e^ |
| Missing | 0 | 0.0 ^a^ | 0 | 0.0 ^b^ | 0 | 0.0 ^c^ | 0 | 0.0 ^d^ | 0 | 0.0 ^e^ |
| Low birth weight | 30 899 | 3.2 ^a^ | 28 430 | 3.2 ^b^ | 1 079 | 4.0 ^c^ | 279 | 3.7 ^d^ | 800 | 4.2 ^e^ |
| Missing | 1 301 | 0.1 ^a^ | 1 224 | 0.1 ^b^ | 36 | 0.1 ^c^ | 11 | 0.1 ^d^ | 25 | 0.1 ^e^ |
| Fetal growth restriction | 33 217 | 3.5 ^a^ | 30 478 | 3.4 ^b^ | 1 172 | 4.4 ^c^ | 277 | 3.6 ^d^ | 895 | 4.7 ^e^ |
| Missing | 1 341 | 0.1 ^a^ | 1 260 | 0.1 ^b^ | 38 | 0.1 ^c^ | 13 | 0.2 ^d^ | 25 | 0.1 ^e^ |
| Birth asphyxia | 31 923 | 3.3 ^a^ | 29 412 | 3.3 ^b^ | 947 | 3.6 ^c^ | 204 | 2.7 ^d^ | 743 | 3.9 ^e^ |
| Missing | 0 | 0.0 ^a^ | 0 | 0.0 ^b^ | 0 | 0.0 ^c^ | 0 | 0.0 ^d^ | 0 | 0.0 ^e^ |
| Umbilical arterial pH <7.1 | 21 669 | 2.3 ^a^ | 20 031 | 2.2 ^b^ | 681 | 2.6 ^c^ | 169 | 2.2 ^d^ | 512 | 2.7 ^e^ |
| Missing | 352 992 | 36.7 ^a^ | 332 090 | 37.0 ^b^ | 9 859 | 37.0 ^c^ | 3 004 | 39.4 ^d^ | 6 855 | 36.0 ^e^ |
| 1 min Apgar score 0-6 | 48 762 | 5.1 ^a^ | 44 984 | 5.0 ^b^ | 1 655 | 6.2 ^c^ | 451 | 5.9 ^d^ | 1 204 | 6.3 ^e^ |
| Missing | 3 506 | 0.4 ^a^ | 3 292 | 0.4 ^b^ | 99 | 0.4 ^c^ | 32 | 0.4 ^d^ | 67 | 0.4 ^e^ |
| 5 min Apgar score 0-6 | 10 032 | 1.0 ^a^ | 9 153 | 1.0 ^b^ | 354 | 1.3 ^c^ | 88 | 1.2 ^d^ | 266 | 1.4 ^e^ |
| Missing | 522 118 | 54.3 ^a^ | 491 638 | 54.7 ^b^ | 13 555 | 50.8 ^c^ | 4 073 | 53.4 ^d^ | 9 482 | 49.8 ^e^ |
| Section | 151 832 | 15.8 ^a^ | 139 372 | 15.5 ^b^ | 5 244 | 19.7 ^c^ | 1 351 | 17.7 ^d^ | 3 893 | 20.4 ^e^ |
| Planned section | 66 059 | 6.9 ^a^ | 60 586 | 6.7 ^b^ | 2 409 | 9.0 ^c^ | 638 | 8.4 ^d^ | 1 771 | 9.3 ^e^ |
| Emergency section | 85 773 | 8.9 ^a^ | 78 786 | 8.8 ^b^ | 2 835 | 10.6 ^c^ | 713 | 9.4 ^d^ | 2 122 | 11.1 ^e^ |
| Missing mode of delivery | 1 478 | 0.2 ^a^ | 1 393 | 0.2 ^b^ | 42 | 0.2 ^c^ | 13 | 0.2 ^d^ | 29 | 0.2 ^e^ |
| Placenta praevia | 2 717 | 0.3 ^a^ | 2 496 | 0.3 ^b^ | 97 | 0.4 ^c^ | 24 | 0.3 ^d^ | 73 | 0.4 ^e^ |
| Placental ablation | 2 154 | 0.2 ^a^ | 2 009 | 0.2 ^b^ | 55 | 0.2 ^c^ | 14 | 0.2 ^d^ | 41 | 0.2 ^e^ |
| ^a^= percentage from total, ^b^= percentage from controls, ^c^= percentage from mothers with confirmed asthma, ^d^=percentage from mothers with untreated, confirmed asthma, ^e^=percentage from mothers with treated, confirmed asthma | | | | | | | | | | |
