# Supplementary material for: Maternal asthma is associated with increased risk of perinatal mortality
Source: PLoS One. 2018 May 18;13(5):e0197593. doi: 10.1371/journal.pone.0197593 (PMC5959067; doi:10.1371/journal.pone.0197593)
Supplement: S3 Table — Unadjusted and adjusted odds ratios. (DOC) [file pone.0197593.s003.doc]

**Table S3. Perinatal outcomes in mothers with confirmed, treated or untreated asthma for singleton live and stillbirths. Unadjusted and adjusted odds ratios.**

|  | **Confirmed asthma vs control** | | | | **Treated vs. untreated. confirmed asthma** | | | | **Untreated. confirmed asthma vs control** | | | | **Treated. confirmed asthma vs. control** | | | |
| --- | --- | --- | --- | --- | --- | --- | --- | --- | --- | --- | --- | --- | --- | --- | --- | --- |
|  | **OR (95% CI)** | ***P*** | **aOR* (95% CI)** | ***P*** | **OR (95% CI)** | ***P*** | **aOR* (95% CI)** | ***P*** | **OR (95% CI)** | ***P*** | **aOR* (95% CI)** | ***P*** | **OR (95% CI)** | ***P*** | **aOR* (95% CI)** | ***P*** |
| Perinatal mortality | 1.24 (1.05, 1.45) | 0.0109 | 1.26 (1.07, 1.48) | 0.0067 | 0.87 (0.62, 1.22) | 0.4125 | 0.93 (0.66, 1.32) | 0.6903 | 1.37 (1.03, 1.82) | 0.032 | 1.33 (1.00, 1.79) | 0.0533 | 1.18 (0.97, 1.44) | 0.0925 | 1.22 (1.01, 1.49) | 0.0447 |
| Premature birth | 1.18 (1.11, 1.25) | <.0001 | 1.18 (1.12, 1.25) | <.0001 | 0.87 (0.77, 0.99) | 0.0271 | 0.87 (0.77, 0.98) | 0.021 | 1.30 (1.17, 1.43) | <.0001 | 1.31 (1.19, 1.45) | <.0001 | 1.13 (1.06, 1.21) | 0.0004 | 1.13 (1.05, 1.21) | 0.0005 |
| Low birth weight | 1.29 (1.21, 1.37) | <.0001 | 1.29 (1.21, 1.37) | <.0001 | 1.15 (1.00, 1.33) | 0.0435 | 1.12 (0.97, 1.29) | 0.1144 | 1.16 (1.03, 1.31) | 0.0141 | 1.19 (1.05, 1.34) | 0.0058 | 1.34 (1.25, 1.44) | <.0001 | 1.33 (1.23, 1.43) | <.0001 |
| SGA | 1.31 (1.23, 1.39) | <.0001 | 1.32 (1.24, 1.40) | <.0001 | 1.31 (1.14, 1.50) | 0.0001 | 1.25 (1.09, 1.44) | 0.0015 | 1.07 (0.95, 1.21) | 0.2461 | 1.12 (0.99, 1.26) | 0.0691 | 1.4 (1.31, 1.5) | <.0001 | 1.39 (1.30, 1.49) | <.0001 |
| Umbilical arterial pH < 7.1 | 1.15 (1.07, 1.24) | 0.0004 | 1.17 (1.08, 1.26) | <.0001 | 1.15 (0.97, 1.38) | 0.1128 | 1.10 (0.92, 1.31) | 0.3027 | 1.04 (0.89, 1.21) | 0.6537 | 1.10 (0.94, 1.28) | 0.2449 | 1.2 (1.09, 1.31) | <.0001 | 1.20 (1.09, 1.31) | 0.0001 |
| 1 min Apgar score 0 to 6 | 1.26 (1.19, 1.32) | <.0001 | 1.26 (1.19, 1.32) | <.0001 | 1.07 (0.96, 1.20) | 0.2206 | 1.01 (0.91, 1.14) | 0.8172 | 1.19 (1.09, 1.31) | 0.0003 | 1.24 (1.13, 1.36) | <.0001 | 1.28 (1.21, 1.36) | <.0001 | 1.26 (1.19, 1.34) | <.0001 |
| 5 min Apgar score 0 to 6 | 1.21 (1.08, 1.34) | 0.0007 | 1.21 (1.09, 1.35) | 0.0005 | 1.13 (0.88, 1.44) | 0.3433 | 1.05 (0.82, 1.35) | 0.6829 | 1.10 (0.89, 1.37) | 0.3629 | 1.15 (0.93, 1.43) | 0.1934 | 1.24 (1.1, 1.41) | 0.0006 | 1.23 (1.09, 1.40) | 0.0009 |
| Urgent section | 1.24 (1.19, 1.29) | <.0001 | 1.23 (1.18, 1.28) | <.0001 | 1.22 (1.11, 1.33) | <.0001 | 1.12 (1.03, 1.23) | 0.0119 | 1.07 (0.99, 1.16) | 0.0731 | 1.13 (1.05, 1.23) | 0.0019 | 1.3 (1.25, 1.37) | <.0001 | 1.27 (1.21, 1.33) | <.0001 |
| Asphyxia | 1.09 (1.02, 1.16) | 0.0126 | 1.07 (1.00, 1.14) | 0.0454 | 1.48 (1.26, 1.73) | <.0001 | 1.32 (1.12, 1.55) | 0.0007 | 0.81 (0.71, 0.93) | 0.0035 | 0.88 (0.77, 1.01) | 0.0774 | 1.2 (1.11, 1.29) | <.0001 | 1.14 (1.06, 1.23) | 0.0007 |
| * Adjusted for maternal age, parity, smoking, socio-economic status, year of birth | | | | | | | | | | | | | | | | |
