# Supplementary material for: Maternal asthma is associated with increased risk of perinatal mortality
Source: PLoS One. 2018 May 18;13(5):e0197593. doi: 10.1371/journal.pone.0197593 (PMC5959067; doi:10.1371/journal.pone.0197593)
Supplement: S4 Table — (DOC) [file pone.0197593.s004.doc]

**Table S4. Perinatal outcomes of treated, confirmed asthma by ATC-groups compared to controls for singleton live and stillbirths.**

|  |  | **Perinatal mortality** | | | **Premature birth** | | | **Low birth weight** | | | **Small for gestational age** | | |
| --- | --- | --- | --- | --- | --- | --- | --- | --- | --- | --- | --- | --- | --- |
|  | **Users (total)** | **N** | **aOR (CI 95%)** | **pval** | **N** | **aOR (CI 95%)** | **pval** | **N** | **aOR (CI 95%)** | **pval** | **N** | **aOR (CI 95%)** | **pval** |
| Any asthma medication | 19 050 | 104 | 1.20 (0.98, 1.46) | 0.0734 | 894 | 1.12 (1.05, 1.20) | 0.001 | 800 | 1.33 (1.24, 1.43) | <.0001 | 895 | 1.40 (1.31, 1.50) | <.0001 |
| β2 agonists | 14 104 | 80 | 1.23 (0.99, 1.54) | 0.0671 | 658 | 1.11 (1.02, 1.20) | 0.0105 | 603 | 1.34 (1.23, 1.45) | <.0001 | 668 | 1.39 (1.29, 1.51) | <.0001 |
| Short-acting β2 agonists | 13 527 | 79 | 1.27 (1.01, 1.59) | 0.0377 | 628 | 1.11 (1.02, 1.20) | 0.0162 | 575 | 1.33 (1.22, 1.45) | <.0001 | 637 | 1.39 (1.28, 1.50) | <.0001 |
| Long-acting β2 agonists | 1 732 | 8 | 0.99 (0.50, 1.99) | 0.9863 | 98 | 1.32 (1.08, 1.63) | 0.0072 | 90 | 1.59 (1.28, 1.97) | <.0001 | 102 | 1.69 (1.38, 2.07) | <.0001 |
| Combination product of long-acting β2 agonists and inhaled glucocorticoids | 4 705 | 26 | 1.14 (0.77, 1.69) | 0.5249 | 218 | 1.09 (0.95, 1.25) | 0.2321 | 195 | 1.26 (1.09, 1.46) | 0.0017 | 220 | 1.34 (1.17, 1.53) | <.0001 |
| Inhaled anticholinergics | 164 | 1 | 1.11 (0.16, 7.98) | 0.9167 | 10 | 1.39 (0.73, 2.63) | 0.3173 | 7 | 1.23 (0.58, 2.64) | 0.5877 | 5 | 0.82 (0.34, 2.01) | 0.6642 |
| Short-acting anticholinergics | 125 | 0 | NA | NA | 7 | 1.27 (0.59, 2.73) | 0.5352 | 6 | 1.38 (0.61, 3.15) | 0.4421 | 3 | 0.63 (0.20, 1.99) | 0.4292 |
| Long-acting anticholinergics | 32 | 0 | NA | NA | 2 | 1.39 (0.33, 5.82) | 0.655 | 1 | 0.85 (0.12, 6.30) | 0.8772 | 2 | 1.71 (0.40, 7.21) | 0.4679 |
| Inhaled glucocorticoids | 13 777 | 64 | 1.03 (0.80, 1.32) | 0.8487 | 613 | 1.06 (0.98, 1.15) | 0.1482 | 584 | 1.35 (1.24, 1.47) | <.0001 | 677 | 1.49 (1.37, 1.61) | <.0001 |
| Xanthines | 285 | 3 | 2.22 (0.71, 6.94) | 0.1707 | 16 | 1.29 (0.78, 2.14) | 0.3212 | 12 | 1.23 (0.69, 2.20) | 0.4844 | 20 | 2.01 (1.27, 3.18) | 0.003 |
| Leukotriene receptor antagonists | 902 | 7 | 1.69 (0.80, 3.55) | 0.1786 | 54 | 1.40 (1.06, 1.84) | 0.0176 | 48 | 1.63 (1.22, 2.18) | 0.0011 | 54 | 1.72 (1.30, 2.27) | 0.0001 |
| Oral glucocorticoids | 2 352 | 16 | 1.43 (0.88, 2.35) | 0.1528 | 141 | 1.42 (1.20, 1.69) | <.0001 | 106 | 1.37 (1.13, 1.67) | 0.0016 | 141 | 1.73 (1.46, 2.06) | <.0001 |
| * Adjusted for maternal age, parity, smoking, socio-economic status, year of birth | | | | | | | | | | | | | |
